# [^161^Tb]Tb-DOTATATE as a potential treatment option for neuroblastoma

**DOI:** 10.1186/s13550-026-01487-9

**Published:** 2026-07-28

**Authors:** Saloni Chopra, Tianqi Xu, Hanna Berglund, Amanda Gustafsson, Marika Nestor

**Affiliations:** Department of Immunology, Genetics and Pathology, Uppsala University, SE-751 85 Uppsala, Sweden

**Keywords:** Neuroblastoma, Terbium-161, Lutetium-177, Radionuclide therapy

## Abstract

**Background:**

Neuroblastoma is an aggressive paediatric malignancy with poor outcomes in high-risk patients despite multimodal therapy. [^177^Lu]Lu-DOTATATE, targeting somatostatin receptor 2 (SSTR2), is under clinical investigation for neuroblastoma. Terbium-161 (^161^Tb) shares chemical characteristics and a similar half-life with lutetium-177 (^177^Lu) but offers advantages through its higher emission of Auger and conversion electrons, which may enhance therapeutic efficacy especially for micrometastases and recurrent disease. This study investigates [^161^Tb]Tb-DOTATATE as a potential complement for treatment of SSTR2-expressing neuroblastoma.

**Results:**

DOTATATE was successfully radiolabelled with ^161^Tb, achieving high radiochemical yield and stability. [^161^Tb]Tb-DOTATATE demonstrated clear SSTR2-specific binding in vitro, as well as biodistribution and tumour uptake comparable to [^177^Lu]Lu-DOTATATE in vivo. Treatment with [^161^Tb]Tb-DOTATATE reduced tumour growth in an activity-dependent manner, and demonstrated stronger early tumour control compared to [^177^Lu]Lu-DOTATATE at comparable administered activities (**p < 0.01 at day 9). Tumour doubling times were longest in the 6 MBq [^161^Tb]Tb-DOTATATE group (29 vs. 18 days in controls), while the 3 MBq [^161^Tb]Tb-DOTATATE group showed similar doubling times to 4 MBq [^177^Lu]Lu-DOTATATE (21 vs. 22 days). This was also reflected in survival, where 6 MBq [^161^Tb]Tb-DOTATATE achieved the greatest effect (50% increase in median survival), followed by 4 MBq [^161^Tb]Tb-DOTATATE (36%), while 3 MBq [^161^Tb]Tb-DOTATATE and 4 MBq [^177^Lu]Lu-DOTATATE showed more modest improvements (23%). All treatment regimens were well tolerated, and no signs of treatment-related toxicity were observed.

**Conclusion:**

[^161^Tb]Tb-DOTATATE demonstrates favourable targeting properties and activity-dependent therapeutic efficacy in preclinical neuroblastoma models without detectable toxicity. These findings encourage us to explore its potential as a complement to [^177^Lu]Lu-DOTATATE, particularly for targeting minimal residual disease and micrometastases, and warrant further preclinical and clinical evaluation.

**Supplementary Information:**

The online version contains supplementary material available at 10.1186/s13550-026-01487-9.

## Introduction

Neuroblastoma is a paediatric cancer form arising from the sympathetic nervous system, mostly originating from the adrenal gland. It primarily affects children below the age of five and is responsible for 10–15% of all paediatric cancer-related deaths [1]. It exhibits a heterogeneous disease profile, ranging from spontaneous regression to aggressive and metastatic disease [2]. Approximately half of cases are classified as high-risk and are associated with a five-year survival rate of 50% despite a vast arsenal of treatment options. The treatment depends on the classification stage and can include surgical resection of the primary tumour, external beam radiotherapy (EBRT), chemotherapy, and high doses of retinoic acid [3]. One prominent reason for recurrence is the presence of micrometastases, which commonly remain unaffected by conventional treatment methods [4].

A promising treatment strategy for metastatic disease is targeted radionuclide therapy (TRT), in which a radionuclide is conjugated to a molecule that binds specifically to the cancer cells, enabling targeted delivery of radiation, even to small tumours that may go undetected in diagnostic scans [5, 6]. [^177^Lu]Lu-DOTATATE ([^177^Lu]Lu-[DOTA^0^,Tyr^3^]octreotate; Lutathera®) is a radiopharmaceutical targeting somatostatin receptor 2 (SSTR2), consisting of ^177^Lu chelated to a somatostatin analogue. It is currently used for the treatment of SSTR2 overexpressing gastroenteropancreatic neuroendocrine tumours [7, 8]. Studies have shown that around 70–90% of neuroblastoma patient samples demonstrate an SSTR2 overexpression, making [^177^Lu]Lu-DOTATATE an attractive treatment option [9–13]. Preclinical studies have shown promising results for treating neuroblastoma with [^177^Lu]Lu-DOTATATE, and early-stage clinical trials (NCT04903899, registered May 19, 2021; NCT03966651, registered April 17, 2023) are currently ongoing [4–6, 14–16].

Lutetium-177 is a beta-emitting radionuclide with a maximum range in tissue of around 2 mm (average 0.7 mm). ^177^Lu is particularly well suited for the treatment of small- to medium-sized tumours and disseminated disease with sufficient target expression, where its β⁻ emissions enable effective cross-fire irradiation. Its release of γ-photons makes it suitable for single-photon emission computed tomography (SPECT) imaging, allowing for dual use of the same radioconjugate. However, its capacity to eradicate isolated tumour cells or microscopic metastases is limited, as the absorbed dose to such small targets may be insufficient to cause lethal DNA damage [17].

Terbium-161 (154 keV) demonstrates similar chemical and physical properties as ^177^Lu (134 keV), including the above-mentioned dual use characteristic, while producing a higher proportion of low-energy Auger and conversion electrons. Importantly, these particles have a shorter tissue range and higher linear energy transfer (LET) per decay, which could potentially be more efficient in treating micrometastases [18]. Preclinical studies have shown that the biodistribution in normal organs is very similar for a molecule labelled with either ^177^Lu or ^161^Tb, but that a higher therapeutic effect can be achieved with the latter [17, 19–21]. Preclinical studies investigating ^161^Tb-labelled DOTATATE and the closely related DOTA-TOC have shown promising results in neuroendocrine tumour models. Importantly, first-in-human clinical studies with [^161^Tb]Tb-DOTA-TOC and [^161^Tb]Tb-DOTA-LM3 have demonstrated the feasibility of ^161^Tb-labelled somatostatin analogues without reported acute adverse reactions, supporting further clinical evaluation of ^161^Tb-based radionuclide therapy [22, 23]. This is further reflected by ongoing clinical trials evaluating ^161^Tb-based radiopharmaceuticals, including evaluation of [^161^Tb]Tb-PSMA in [^177^Lu]Lu-PSMA naïve prostate cancer patients and [^161^Tb]Tb-DOTATATE in metastatic neuroendocrine tumours (NCT07441837, registered June 01, 2026) and (NCT07404176, registered March 01, 2026) [24, 25].

The aim of this study was to evaluate, for the first time, the potential of [^161^Tb]Tb-DOTATATE in a preclinical neuroblastoma model. We compared its cellular binding, in vivo biodistribution, tumour uptake, toxicity and therapeutic efficacy to those of [^177^Lu]Lu-DOTATATE. Our findings provide the first evidence supporting [^161^Tb]Tb-DOTATATE as a promising candidate for improved TRT in SSTR2-positive neuroblastoma.

## Materials and methods

### Cell lines

The U2OS osteosarcoma cell line transfected with SSTR2 (U2OS-SSTR2) was developed and kindly provided by Professor Julie Nonnekens at Erasmus MC in Rotterdam and cultured in Dulbecco’s Minimal Essential Media (DMEM) (Gibco-Life Technologies/Thermo Fisher, Waltham, MA, USA) [26]. IMR-32-luc cells were kindly provided by Prof. Fredrik Swartling and Dr. Tobias Bergström, Uppsala University [27]. Tumour growth was monitored by calliper measurements 2–3 times per week. Luciferase expression was originally introduced to facilitate in vivo monitoring in cases where tumour growth was difficult to assess by calliper measurements alone. In the present study, tumour growth could be reliably monitored by calliper measurements alone, and additional in vivo imaging was therefore not warranted. Cells were cultured in a RPMI-1640 (Gibco-Life Technologies/Thermo Fisher Waltham, MA, USA). All media was supplemented with 10% fetal bovine serum and 1% antibiotics (100 IU penicillin and 100 mg/mL streptomycin, Sigma Aldrich, MO, USA). The media for IMR-32-luc was further supplemented with 2 ug/mL Puromycin (10 mg/mL Gibco, Life Technologies, Waltham, MA, USA) as selection antibiotic.

### Radioconjugate preparation and stability

Radiolabelling was performed by mixing DOTATATE (1.5 ug; 1 mg/mL in metal free water) (Bachem, Bubendorf, Switzerland) with approximately 60 MBq of non-carrier added [^161^Tb]TbCl_3_ (0.05 M HCl) (supplied by the PRISMAP facility POLATOM, Poland) and a labelling buffer containing sodium acetate (50 mM) and sodium ascorbate (25 mM) at pH 5, using a 1:2 (v/v) ratio of [^161^Tb]TbCl_3_ to labelling buffer with total reaction volume roughly 35 µL. The mixture was heated at 80 °C for 30 min and the radiochemical yield was analysed using instant thin layer chromatography (ITLC) using 0.1 M sodium citrate (pH 5.5) as the mobile phase. Stability of the radioconjugate was assessed in EDTA, mouse serum and PBS at 37 °C for 48 h using ITLC. [^177^Lu]Lu-DOTATATE was radiolabelled as previously described by Lundsten et al. [28], using the same conditions as mentioned above.

### Binding specificity of [^161^Tb]Tb-DOTATATE and [^177^Lu]Lu-DOTATATE

Cellular uptake and binding specificity of [^161^Tb]Tb-DOTATATE and [^177^Lu]Lu-DOTATATE was assessed in U2OS-SSTR2 and IMR-32-luc cells. 3 × 10^5^ cells/well were seeded in a 24 well plate and incubated overnight at 37°C. DOTATATE labelled with either ^177^Lu or ^161^Tb was added to a final concentration of 10 nM to all wells, and 1 µM unlabelled DOTATATE was added to the blocked control wells prior to adding radioactivity. After 4 h of incubation at 37 °C, the media was removed, and the unbound compound was washed away with PBS before the cells were trypsinized, collected and counted. Cell-associated radioactivity was measured using a gamma counter (1480 Wizard 3″, Wallace, Finland) and the normalized uptake for the different groups was compared.

### LigandTracer binding assay

Binding affinity of [^161^Tb]Tb-DOTATATE was assessed using LigandTracer White and Grey (Ridgeview Instruments AB, Uppsala, Sweden). 1 × 10^6^ IMR-32-luc cells were seeded on a 10 cm^2^ polydopamine spot-coated petri dish and 5 × 10^5^ U2OS-SSTR2 cells were seeded on a tilted 10 cm^2^ petri dish according to the manufacturer’s instructions. After 48 h incubation at 37 °C, the media was replaced with 3 mL fresh CO_2_ independent media (Gibco/Thermo Fisher), and a baseline measurement without added compound was performed. After 20 min, [^161^Tb]Tb-DOTATATE was added to a concentration of 3 nM. The association was allowed to run for approximately two hours before the concentration was raised to 10 nM. Finally, all media was removed and the petri dish was washed once with 3 mL CO_2_ independent media before 3 mL fresh media was added and the dissociation phase was run overnight. The obtained data was analysed using TraceDrawer software v.1.10.1 (Ridgeview Instruments AB).

### Xenograft establishment

All animal experiments complied with Swedish legislation and were approved by the Uppsala Committee of Animal Research Ethics (Permit numbers: 10966/2020 and 5.8.18–05467/2025). Female athymic nude mice (Hsd:AthymicNude-Foxn1nu, Envigo, Huntingdon, UK), aged five weeks and weighing 20–25 g, were included in the study. Tumour-bearing mice were generated by subcutaneous injection of 5 × 10^6^ IMR-32-luc cells suspended in 100 µL of a 1:1 mixture of PBS and Matrigel (Corning Life Sciences, Corning, NY, USA) into the right hind flank. All animals were maintained under standard laboratory conditions with ad libitum access to food and water. The mice were weighed at least once per week, and when tumours had formed, the width and length were measured 2 – 3 times per week using a digimatic calliper (Mitutoyo, Sweden). Tumour volume was calculated as (ab^2^)/2, where a is the length and b is the width. In the biodistribution and therapy studies, treatment was initiated 27 and 21 days after tumour inoculation, respectively, when mean tumour volumes were approximately 350 mm^3^ and 120 mm^3^. Tumour volumes did not differ significantly between treatment groups at study start. A tumour volume equal to or above 1000 mm^3^ or weight loss of more than 15% from treatment start were considered humane endpoints. Euthanasia was performed through intraperitoneal injection of Ketamine (10 mg/mL) and Xylazine (1 mg/mL) followed by heart puncture.

### IHC staining

Tumour samples were prepared as described previously [29]. Paraffin sections were deparaffinised, rehydrated and subjected to heat-induced antigen retrieval. Sections were blocked and incubated with a primary antibody directed against SSTR2 (Abcam ab134152 diluted 1/500) and visualized with DAB substrate according to manufacturer´s protocol for Vectastain ABC Elite kits (Vector Labs, SK-4105). Sections were counterstained in haematoxylin and eosin, and then mounted.

### [^161^Tb]Tb-DOTATATE and [^177^Lu]Lu-DOTATATE biodistribution

Biodistribution of DOTATATE labelled with either ^161^Tb or ^177^Lu was assessed in 12 mice bearing IMR-32-luc tumours. Mice were intravenously injected with 100 µL containing 500 kBq (0.15 µg; 0.1 nmol DOTATATE) of either [^161^Tb]Tb-DOTATATE or [^177^Lu]Lu-DOTATATE, diluted in 0.9% NaCl (saline). [^161^Tb]Tb-DOTATATE–treated mice were euthanized at 4 h (n = 4) or 24 h (n = 4), and [^177^Lu]Lu-DOTATATE–treated mice at 24 h post-injection (p.i.) (n = 4). Tumour, blood, kidneys, lungs, liver, and other selected organs were collected, weighed, and measured for activity using a gamma counter. The uptake, expressed as percentage injected activity per gram tissue (%IA/g), was then calculated.

### [^161^Tb]Tb-DOTATATE and [^177^Lu]Lu-DOTATATE therapy

Mice bearing IMR-32-luc tumours were divided into five groups and injected intravenously with either 100 µL of saline (n = 10), 3 MBq [^161^Tb]Tb-DOTATATE (n = 9), 4 MBq [^161^Tb]Tb-DOTATATE (n = 9), 6 MBq [^161^Tb]Tb-DOTATATE (n = 9), or 4 MBq [^177^Lu]Lu-DOTATATE (n = 9). A group of tumour-free littermate controls (n = 3) were maintained alongside the treated mice to provide reference values for assessing potential treatment-related toxicity. Tumour size and body weight were monitored over time until the study endpoint, set as 100 days or until all mice are taken down due to tumour burden. Three mice (two in the 4 MBq [^161^Tb]Tb-DOTATATE group and one in the saline group) were excluded from efficacy and tumour growth analyses due to failure of tumour establishment, but were retained for hematologic analyses. Haematologic toxicity was evaluated by serial blood sampling (tail vein, 20 µL per sample) and analysed using a veterinary haematology analyser (Exigo H400, Boule, Sweden). At the end of treatment, kidneys and liver were collected and weighed.

### Statistical analysis

Statistical analysis was performed with Prism 10 (Graphpad, CA). For in vitro analyses, statistical significance was assessed using unpaired t-test and in vivo analyses were performed using one-way ANOVA. Statistical significance is presented as not significant (n.s., p ≥ 0.05), * (p < 0.05), ** (p < 0.01), *** (p < 0.001) and **** (p < 0.0001). An exponential (Malthusian) growth model was applied to perform the non-linear fit of tumour growth curves in the therapy study. Tumour growth delay (TGD) was defined as the difference between the mean time required for tumours in treated group and control group to reach twice their initial volume:

$$TGD={T}_{treated}-{T}_{control}$$

where *T* represents the mean time required for tumours to reach twice their volume at treatment start.

## Results

### Labelling of [^161^Tb]Tb-DOTATATE results in high radiochemical yield and purity

Radiolabelling of DOTATATE with ^161^Tb was performed and analysed by ITLC using 0.1 M sodium citrate (pH 5.5) as the mobile phase. The reaction consistently produced a high radiochemical yield, averaging > 97%. Stability was further assessed in EDTA solution, mouse serum and PBS at 37 °C for up to 48 h post-labelling. Radiochemical purity remained high above 90% under all conditions, with the exception of EDTA, where a slight reduction to 85% was observed at 24 h.

### Binding specificity and affinity of [^161^Tb]Tb-DOTATATE

**Fig. 1:**
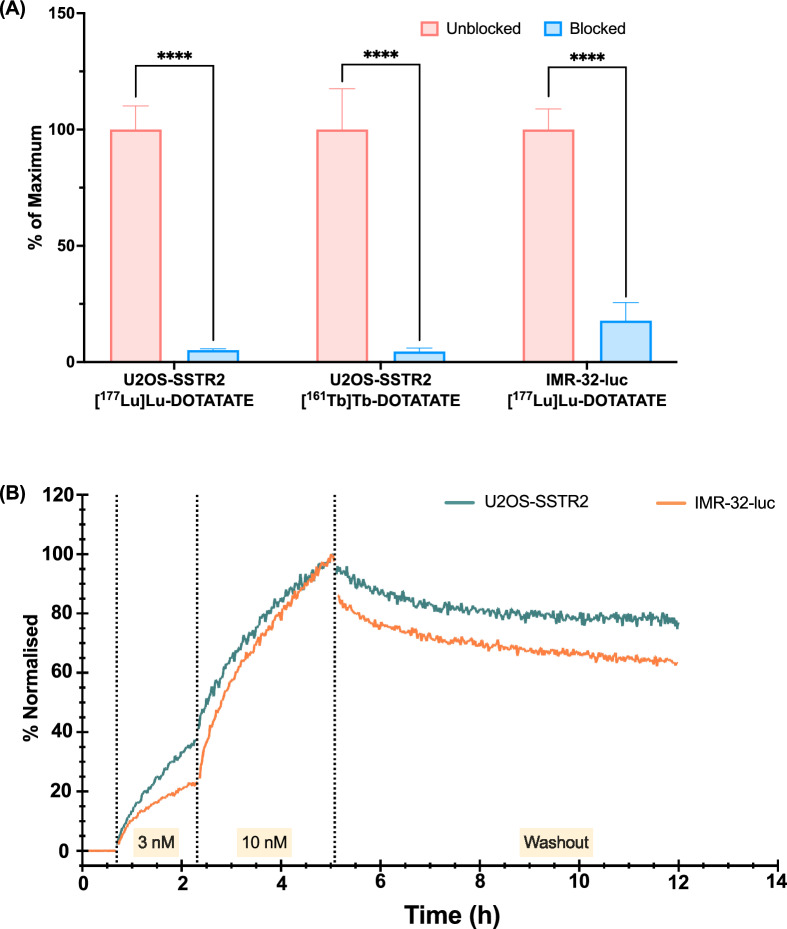
In vitro validation of [^161^Tb]Tb-DOTATATE and [^177^Lu]Lu-DOTATATE on U2OS-SSTR2 and IMR-32-luc cells (**A**) Cellular uptake assay assessing binding specificity and receptor presence. Uptake of radiolabelled DOTATATE was measured in the absence and presence of excess unlabelled DOTATATE. Specific binding of both [^177^Lu]Lu-DOTATATE and [^161^Tb]Tb-DOTATATE was confirmed in U2OS-SSTR2 cells, while SSTR2 expression in IMR-32-luc cells was demonstrated using [^177^Lu]Lu-DOTATATE. Data were normalized to the unblocked condition for each cell line (mean ± SD, n = 4; **** p < 0.0001). (**B**) Real-time binding of [^161^Tb]Tb-DOTATATE measured using LigandTracer in IMR-32-luc (orange) and U2OS-SSTR2 (green) cells at room temperature, normalized to maximum binding

Specific binding of [^161^Tb]Tb-DOTATATE was first evaluated in a receptor specificity assay using U2OS-SSTR2 cells and directly compared with [^177^Lu]Lu-DOTATATE. Uptake of both radioconjugates was efficiently blocked by an excess of unlabelled DOTATATE, confirming SSTR2-specific binding (Fig. 1 A). To further confirm target expression in the neuroblastoma model, [^177^Lu]Lu-DOTATATE was evaluated in IMR-32-luc cells, where a similar blocking pattern was observed, supporting the presence of SSTR2 expression in these cells (Fig. 1 A). Real-time binding kinetics of [^161^Tb]Tb-DOTATATE were subsequently assessed using LigandTracer in both U2OS-SSTR2 and IMR-32-luc cells (Fig. 1 B). U2OS-SSTR2 cells provide a transfected model with high and relatively homogeneous SSTR2 expression, whereas IMR-32-luc cells represent a neuroblastoma model with endogenous SSTR2 expression. Comparable binding profiles were observed in both cell lines, although a slower dissociation was observed in U2OS-SSTR2 cells, consistent with the higher receptor density of this model. The equilibrium dissociation constant (K_D_), determined in IMR-32-luc cells using a 1:2 binding model, was in the low-nanomolar range, consistent with previous reports for [^177^Lu]Lu-DOTATATE [16].

### [^161^Tb]Tb-DOTATATE demonstrates in vivo targeting and a similar biodistribution profile as [^177^Lu]Lu-DOTATATE

**Fig. 2:**
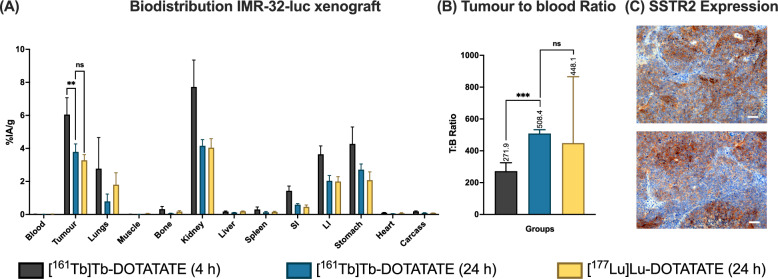
In vivo validation of [^161^Tb]Tb-DOTATATE in IMR-32-luc xenograft models (**A**) Biodistribution data at 4 h (n = 4) and 24 h (n = 4) post-injection (p.i.) of [^161^Tb]Tb-DOTATATE and at 24 h p.i. of [^177^Lu]Lu-DOTATATE (n = 4) in IMR-32-luc xenograft-bearing mice. Error bars = SD. (**B**) Tumour-to-blood ratios for [^161^Tb]Tb-DOTATATE at 4 (n = 4) and 24 h p.i. (n = 4) and [^177^Lu]Lu-DOTATATE at 24 h p.i. (n = 4). Error bars = SD. (**C**) Representative immunohistochemistry images of SSTR2 expression in IMR-32-luc xenograft tumours acquired at 20 × magnification

Biodistribution studies demonstrated comparable uptake patterns, expressed as %IA/g, for [^161^Tb]Tb-DOTATATE and [^177^Lu]Lu-DOTATATE at 24 h p.i., with the highest uptake observed in tumours, kidneys and the gastrointestinal tract (Fig. 2 A, B; Tables 1 and 2). These findings are in line with previous [^177^Lu]Lu-DOTATATE biodistribution data in IMR-32 xenografts [30]. A numerical difference in lung uptake between [^161^Tb]Tb-DOTATATE and [^177^Lu]Lu-DOTATATE was observed at 24 h p.i., with higher mean uptake in the [^177^Lu]Lu-DOTATATE group. However, the difference did not reach statistical significance and was associated with inter-animal variability. Tumour uptake of [^161^Tb]Tb-DOTATATE was 6.0 ± 1.0%IA/g and 3.8 ± 0.5%IA/g at 4 and 24 h p.i. respectively (mean ± SD), indicating sustained tumour retention over time. Uptake for all the organs are shown in Table 1 and Tumour-to-organ ratios are shown in Table 2. Generally, uptake in other non-target tissues was low, with minimal levels in blood and muscle. Tumour-to-blood ratios exceeded 200 at 4 h p.i. and increased further at 24 h (Fig. 2 B, Table 2). Immunohistochemistry confirmed moderate SSTR2 expression in IMR-32-luc xenografts, although staining intensity suggested some degree of heterogeneity across tumour regions (Fig. 2 C).

**Table 1:** In vivo biodistribution of [^161^Tb]Tb-DOTATATE and [^177^Lu]Lu-DOTATATE, expressed as %IA/g (mean ± SD), in IMR-32-luc xenografts

Organs	[^161^Tb]Tb-DOTATATE		[^177^Lu]Lu-DOTATATE
	4 h p.i	24 h p.i	24 h p.i
Blood	0.0 ± 0.0	0.0 ± 0.0	0.0 ± 0.0
Tumour	6.0 ± 1.0	3.8 ± 0.5	3.3 ± 0.3
Lungs	2.8 ± 1.9	0.8 ± 0.4	1.8 ± 0.7
Muscle	0.0 ± 0.0	0.00 ± 0.0	0.1 ± 0.0
Bone	0.3 ± 0.2	0.1 ± 0.0	0.2 ± 0.1
Kidney	7.7 ± 1.6	4.2 ± 0.4	4.0 ± 0.5
Liver	0.2 ± 0.04	0.1 ± 0.0	0.2 ± 0.0
Spleen	0.3 ± 0.1	0.1 ± 0.0	0.2 ± 0.0
Small intestine	1.4 ± 0.3	0.6 ± 0.1	0.5 ± 0.1
Large intestine	3.6 ± 0.5	2.0 ± 0.3	2.0 ± 0.3
Stomach	4.3 ± 1.0	2.7 ± 0.3	2.1 ± 0.5
Heart	0.1 ± 0.0	0.1 ± 0.0	0.1 ± 0.0
Carcass	0.2 ± 0.0	0.1 ± 0.0	0.1 ± 0.0

**Table 2:** Tumour-to-organ ratio from In vivo biodistribution of [^161^Tb]Tb-DOTATATE and [^177^Lu]Lu-DOTATATE, 4 and 24 h p.i. (mean ± SD) in IMR-32-luc xenografts

Tumour (T) to Organ ratios	[^161^Tb]Tb-DOTATATE		[^177^Lu]Lu-DOTATATE
	4 h p.i	24 h p.i	24 h p.i
Blood	271.9 ± 52.7	508.4 ± 24.1	448.1 ± 417.6
Lungs	4.5 ± 5.1	9.6 ± 11.8	2.1 ± 0.9
Muscle	501.3 ± 172.2	1760.9 ± 1942.5	69.2 ± 22.5
Bone	25.1 ± 16.2	67.0 ± 9.6	19.6 ± 6.8
Kidney	0.8 ± 0.2	0.9 ± 0.2	0.8 ± 0.1
Liver	35.8 ± 10.5	32.5 ± 6.1	17.7 ± 3.8
Spleen	22.3 ± 6.4	29.3 ± 5.7	21.3 ± 4.3
Small intestine	4.3 ± 1.0	6.4 ± 0.9	7.7 ± 2.9
Large intestine	1.7 ± 0.4	1.9 ± 0.5	1.7 ± 0.4
Stomach	1.4 ± 0.2	1.4 ± 0.2	1.7 ± 0.4
Heart	65.3 ± 24.5	72.7 ± 6.1	60.5 ± 40.7
Carcass	34.0 ± 10.2	40.7 ± 5.3	36.8 ± 4.2

### Reduced tumour growth and prolonged survival with [^161^Tb]Tb-DOTATATE

Tumour growth, therapeutic efficacy and toxicity of [^161^Tb]Tb-DOTATATE and [^177^Lu]Lu-DOTATATE was assessed in mice bearing IMR-32-luc tumour xenografts. The mice were intravenously injected with either placebo (saline), 3 MBq [^161^Tb]Tb-DOTATATE, 4 MBq [^161^Tb]Tb-DOTATATE, 6 MBq [^161^Tb]Tb-DOTATATE or 4 MBq [^177^Lu]Lu-DOTATATE and followed until a humane endpoint or the predefined study endpoint (100 days) was reached. In the 6 MBq [^161^Tb]Tb-DOTATATE group, one mouse exhibited a markedly slower tumour doubling time compared to the others and was considered an outlier for tumour doubling time calculations. It was therefore excluded from the non-linear exponential growth analysis used to calculate tumour doubling time, but included in all other analyses.

**Fig. 3:**
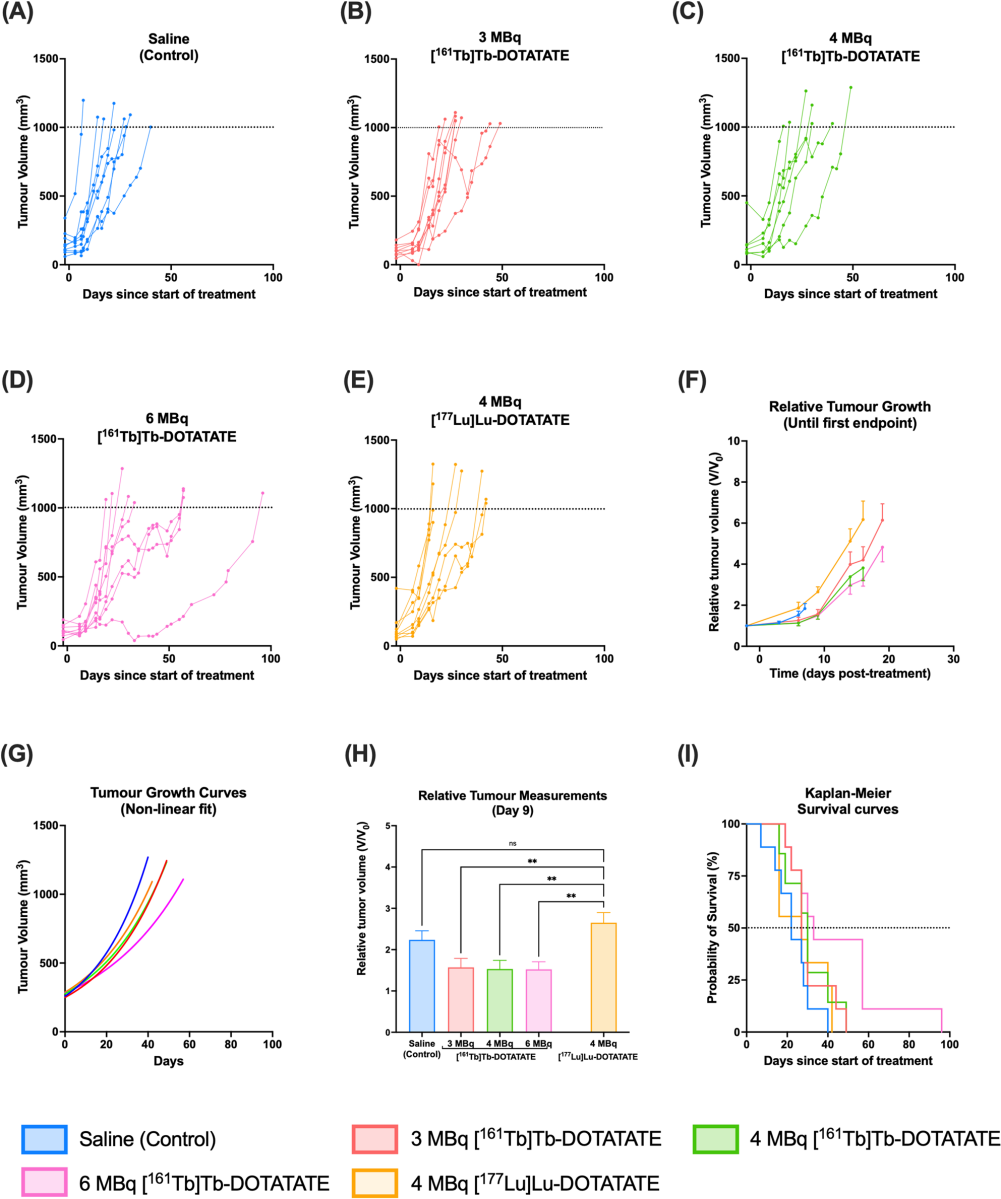
Tumour growth in IMR-32-luc xenografts treated with [^161^Tb]Tb-DOTATATE or [^177^Lu]Lu-DOTATATE. (**A**–**E**) Individual tumour growth curves in IMR-32-luc xenograft-bearing mice receiving (**A**) saline (control), (**B**) 3 MBq [^161^Tb]Tb-DOTATATE, (**C**) 4 MBq [^161^Tb]Tb-DOTATATE, (**D**) 6 MBq [^161^Tb]Tb-DOTATATE, and (**E**) 4 MBq [^177^Lu]Lu-DOTATATE. (**F**) Baseline-corrected relative tumour growth for all groups up to the first euthanized animal in each group. (**G**) Corresponding nonlinear fit of tumour growth for each group (N = 42; n ≥ 7). (**H**) Bar graph showing baseline-corrected relative tumour volumes on day 9 in the study (N = 43; n ≥ 7; error bars = SEM). [^161^Tb]Tb-DOTATATE induced stronger early tumour growth inhibition than [^177^Lu]Lu-DOTATATE (**, p < 0.01 at day 9). Statistical significance was determined using one-way ANOVA. (**I**) Kaplan–Meier survival curves for each treatment group. [^161^Tb]Tb-DOTATATE treatment conferred a dose-dependent survival benefit compared to controls, with the 6 MBq group showing the longest overall survival (N = 43; n ≥ 7)

Treatment with [^161^Tb]Tb-DOTATATE reduced tumour growth in an activity-dependent manner, and demonstrated stronger early tumour control compared to [^177^Lu]Lu-DOTATATE at comparable administered activities. At day 9 post-treatment, relative tumour volumes in all [^161^Tb]Tb-DOTATATE groups (3, 4, and 6 MBq) were significantly smaller than in the 4 MBq [^177^Lu]Lu-DOTATATE group (**p < 0.01) (Fig. 3 H). Tumour volumes in the 4 MBq [^177^Lu]Lu-DOTATATE group were comparable to those of saline injected controls, indicating limited early treatment effect at this injected activity. By day 16 (Supplementary Fig. 1), tumour growth had however progressed in all groups, and the differences between [^161^Tb]Tb-DOTATATE and [^177^Lu]Lu-DOTATATE were reduced. At this time point, only the 6 MBq [^161^Tb]Tb-DOTATATE group remained significantly different from 4 MBq [^177^Lu]Lu-DOTATATE (*p < 0.05), consistent with an activity-dependent therapeutic effect of ^161^Tb. These findings suggest that [^161^Tb]Tb-DOTATATE provides stronger early tumour control than [^177^Lu]Lu-DOTATATE in these experimental settings.

Tumour growth over time is shown in Fig. 3 A-E and Table 3. In the control group, rapid tumour progression was observed, with most tumours reaching endpoint volumes within 14–23 days and a mean tumour doubling time of 18 days (95% CI: 14–23). Treatment with 3 MBq [^161^Tb]Tb-DOTATATE resulted in a modest increase in tumour doubling time to 21 days (95% CI: 17–27), while 4 MBq extended this to 23 days (95% CI: 18–31). The 6 MBq [^161^Tb]Tb-DOTATATE group showed a longer mean doubling time of 29 days (95% CI: 24–36), with several tumours exhibiting delayed progression beyond 40 days. Mice treated with 4 MBq [^177^Lu]Lu-DOTATATE had a mean tumour doubling time of 22 days (95% CI: 17–29). Overall, these findings suggest an activity-dependent trend in tumour growth delay for [^161^Tb]Tb-DOTATATE. Median survival times reflected the tumour growth data and were extended in all treatment groups compared to the saline control (22 days) (Fig. 3 I, Table 3). Mice treated with 3 MBq [^161^Tb]Tb-DOTATATE and 4 MBq [^177^Lu]Lu-DOTATATE both showed a median survival of 27 days (23% increase), while 4 MBq [^161^Tb]Tb-DOTATATE prolonged survival to 30 days (36% increase). The greatest effect was observed in the 6 MBq [^161^Tb]Tb-DOTATATE group, with a median survival of 33 days (50% increase), which was significantly longer than in the control group. Overall, these results support an activity-dependent improvement in survival with [^161^Tb]Tb-DOTATATE.

**Table 3:** In vivo therapy therapeutic efficacy of [^161^Tb]Tb-DOTATATE and [^177^Lu]Lu-DOTATATE in IMR-32-luc xenografts

	Saline (control)	3 MBq [^161^Tb]Tb-DOTATATE	4 MBq [^161^Tb]Tb-DOTATATE	6 MBq [^161^Tb]Tb-DOTATATE	4 MBq [^177^Lu]Lu-DOTATATE
Tumour doubling time (days, 95% CI)	18 (14 to 23)	21 (17 to 27)	23 (18 to 31)	29 (24 to 35)	22 (17 to 29)
First mouse euthanized (day)	7	22	16	19	16
Last mouse euthanized (day)	40	44	49	96	42
Median survival (days, 95% CI)	22 (7–30)	27 (19–44)	30 (16–40)	33 (19–57)	27 (16 +)
TGD	0	3.9	4.3	8.6	0

Tumour growth delay (TGD) analysis (Table 3) further supported an activity-dependent therapeutic effect of [^161^Tb]Tb-DOTATATE in IMR-32-luc xenografts. TGD increased from 3.9 days at 3 MBq to 4.3 days at 4 MBq and 8.6 days at 6 MBq, while no delay was observed for 4 MBq [^177^Lu]Lu-DOTATATE (0 days), comparable to the control group. These results suggest a possible advantage of [^161^Tb]Tb-DOTATATE over [^177^Lu]Lu-DOTATATE at equivalent administered activity.

**Fig. 4:**
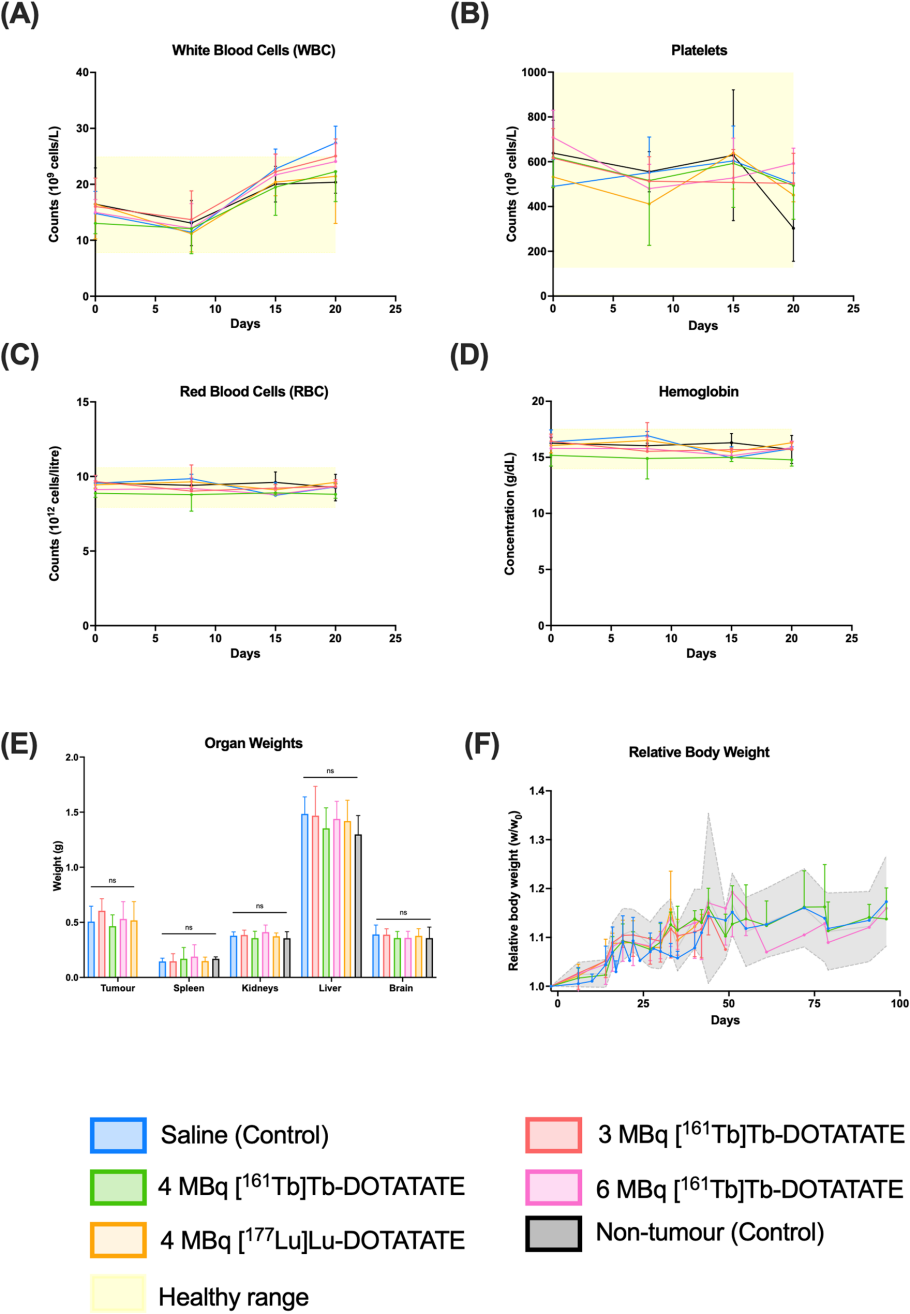
Toxicity assessment following [^161^Tb]Tb-DOTATATE and [^177^Lu]Lu-DOTATATE treatment. Serial blood sampling and body weight monitoring were performed to evaluate potential treatment-related toxicity (Error bars = SEM, n ≥ 7). (**A**) White blood cell counts, (**B**) platelet counts, (**C**) red blood cell counts, and (**D**) haemoglobin concentrations remained within the range of healthy non-tumour controls throughout the study period. (**E**) Post-mortem organ weights (tumour, spleen, kidneys, liver, brain) did not reveal treatment-related differences compared to controls. (**F**) Relative body weights were stable in all groups, and no abnormal clinical signs were observed

Serial blood sampling and body weight monitoring were performed to evaluate potential treatment-related toxicity (Fig. 4). Across all treatment groups, including [^161^Tb]Tb-DOTATATE and [^177^Lu]Lu-DOTATATE, red blood cell, white blood cell, and haemoglobin levels remained comparable to non-tumour bearing healthy controls throughout the study period. Body weights were stable in all groups, and no abnormal clinical signs were observed. Post-mortem organ weights did not reveal any treatment-related differences compared to healthy controls. Together, these findings indicate that none of the tested regimens induced measurable systemic toxicity under the conditions used in this study.

## Discussion

High-risk neuroblastoma remains a major therapeutic challenge in paediatric oncology, with survival rates for relapsed disease remaining dismal despite aggressive multimodal treatment. Minimal residual disease and micrometastases are particularly difficult to eradicate with existing therapies, highlighting the need for innovative approaches that can selectively target and destroy disseminated tumour cells. SSTR2-targeted TRT using [^177^Lu]Lu-DOTATATE has shown encouraging results in neuroblastoma, but its β⁻ emissions may not be optimal for subcellular and microscopic disease. In this context, the present study provides the first preclinical evaluation of [^161^Tb]Tb-DOTATATE in neuroblastoma, assessing whether DOTATATE can be successfully labelled with ^161^Tb while preserving its targeting properties and achieving antitumour effects comparable to [^177^Lu]Lu-DOTATATE, with potential future relevance for micrometastatic disease.

In the present study, we demonstrate that DOTATATE can be efficiently labelled with ^161^Tb with high yield and preserved SSTR2 binding affinity, comparable to its ^177^Lu-labelled counterpart (Fig. 1). Biodistribution profiles and tumour-to-organ ratios in vivo were also highly similar between the two radioconjugates (Fig. 2, Tables 1, 2). These findings align with prior studies in other SSTR2-positive tumour models, where ^161^Tb-labelled peptides exhibited pharmacokinetics similar to their ^177^Lu analogues [17, 21], reinforcing that substitution of the radionuclide does not adversely affect targeting properties. The current data therefore support the view that ^161^Tb can be introduced into the DOTATATE scaffold without losing the properties that make the construct attractive for receptor-targeted therapy.

Tumour uptake of both radioconjugates in IMR-32-luc xenografts was approximately 4%IA/g (Fig. 2, Table 2), slightly lower than the ~ 6%IA/g previously reported in IMR-32 tumours [31]. This difference is consistent with slightly reduced and more heterogeneous SSTR2 expression noted in IMR-32-luc xenografts in the present study, as indicated by the IHC data (Fig. 2 C), and aligns with our previous observations in this model [27]. Uptake in the gastrointestinal tract was somewhat higher than previously reported, which may reflect differences in experimental conditions, including the mouse model used. Despite moderate antigen expression, tumour-to-organ ratios remained favourable, indicating sufficient target engagement for therapeutic evaluation.

In subsequent therapy experiments, [^161^Tb]Tb-DOTATATE demonstrated activity-dependent tumour growth inhibition and survival benefit without signs of acute toxicity. At comparable administered activities, stronger early tumour control was observed with [^161^Tb]Tb-DOTATATE compared to [^177^Lu]Lu-DOTATATE (Fig. 3, Table 3), particularly at early time points (**p < 0.01 at day 9) (Fig. 3 H), suggesting that ^161^Tb may confer an early therapeutic advantage even in a model with heterogeneous and moderate SSTR2 expression. In addition, some [^161^Tb]Tb-DOTATATE-treated animals, particularly in the higher-activity groups, exhibited prolonged periods of tumour growth stabilisation following treatment. While the limited group sizes preclude firm conclusions, this observation is consistent with the overall therapeutic trends observed in the study. These results are also consistent with previous preclinical studies in other tumour types, where ^161^Tb-labelled agents outperformed ^177^Lu analogues in controlling small-volume disease, both in vitro and in vivo [17, 22, 23]. Given the highly similar biodistribution profiles observed for [^161^Tb]Tb-DOTATATE and [^177^Lu]Lu-DOTATATE, the improved therapeutic response observed in the present study is more likely attributed to differences in radiation quality rather than differences in tumour targeting. Specifically, the higher emission of short-range conversion and Auger electrons from ^161^Tb, which may enhance energy deposition in small tumour clusters or residual microscopic disease where β⁻ particles from ^177^Lu are less effective, suggesting a potential advantage for early tumour control in such settings. In addition to enhanced DNA damage, these emissions may contribute to increased damage to other cellular structures following radioconjugate internalization, potentially improving tumour cell kill despite comparable tumour uptake. While the present xenograft model was not designed to directly assess effects in micrometastatic disease, the observed therapeutic advantage suggests that the unique emission profile of ^161^Tb may provide benefits beyond those expected from absorbed dose alone.

Notably, these differences were observed at relatively low administered activities, where [^177^Lu]Lu-DOTATATE showed limited efficacy. Under these conditions, [^161^Tb]Tb-DOTATATE demonstrated measurable tumour growth delay and survival benefit, and in some settings comparable outcomes were achieved at lower activity levels. While these findings suggest a potential advantage of ^161^Tb, they should be interpreted with caution, as the study was not designed to establish dose equivalence or optimal therapeutic regimens.

Importantly, all treatment regimens were well tolerated, with no detectable haematological toxicity or adverse effects on body weight or organ parameters (Fig. 4). This supports that the enhanced efficacy observed with [^161^Tb]Tb-DOTATATE was not associated with increased acute toxicity under the conditions tested.

Several limitations should be considered when interpreting this study. First, the in vivo experiments were conducted in a single subcutaneous human neuroblastoma model in immunodeficient mice with moderate and heterogeneous SSTR2 expression, which does not fully reflect the biological diversity of neuroblastoma in humans. At the same time, the endogenous and heterogeneous receptor expression observed in this model may better reflect the variability encountered in clinical neuroblastoma than highly transfected receptor-overexpressing models. Future studies should assess [^161^Tb]Tb-DOTATATE in additional neuroblastoma models with varying levels of SSTR2 expression. In addition, orthotopic, metastatic, or patient-derived xenograft models may provide valuable complementary information. Second, the comparison relied on single, relatively low administered activities, selected to explore potential differences between radionuclides rather than to define optimal therapeutic conditions. As a result, the optimal activity range, fractionation strategies, and dose equivalence between ^161^Tb and ^177^Lu remain to be established. Future studies should therefore include extended follow-up, detailed dosimetric analyses, and evaluation across multiple neuroblastoma models. Dose-escalation and fractionation studies will be important to define the therapeutic window and to assess efficacy at clinically relevant activity levels. It will also be important to determine whether the observed advantages of ^161^Tb persist in real clinical settings.

## Conclusion

In conclusion, we have for the first time assessed [^161^Tb]Tb-DOTATATE as a potential TRT for neuroblastoma. We show that it can be prepared with high stability and specificity, with preserved target binding, biodistribution, and in vivo tumour targeting. Therapy studies demonstrated activity-dependent effects, achieving comparable or superior efficacy to [^177^Lu]Lu-DOTATATE without observed toxicity. These findings support further investigation of ^161^Tb–based radionuclide therapy, particularly for targeting minimal residual disease and micrometastases.

## Supplementary Information

Supplementary Material 1 Bar graph showing baseline-corrected relative tumour volumes on day 16 (n ≥ 7; error bars = SEM). [^161^Tb]Tb-DOTATATE induced stronger early tumour growth inhibition than [^177^Lu]Lu-DOTATATE, most pronounced at day 9 (**, p < 0.01). By day 16, tumour growth had however progressed in all groups, and only the 6 MBq [^161^Tb]Tb-DOTATATE group remained significantly different from the 4 MBq [177Lu]Lu-DOTATATE (*p < 0.05)

## Data Availability

All data generated or analysed during this study are included in this published article and its supplementary information files. Additional data are available from the corresponding author on reasonable request.
