# Supplementary figures and images for: [161Tb]Tb-DOTATATE as a potential treatment option for neuroblastoma
Source: EJNMMI Res. 2026 Jul 28;16:116. doi: 10.1186/s13550-026-01487-9 (PMC13415435; doi:10.1186/s13550-026-01487-9)

# Relative Tumour Measurements (Day 16)

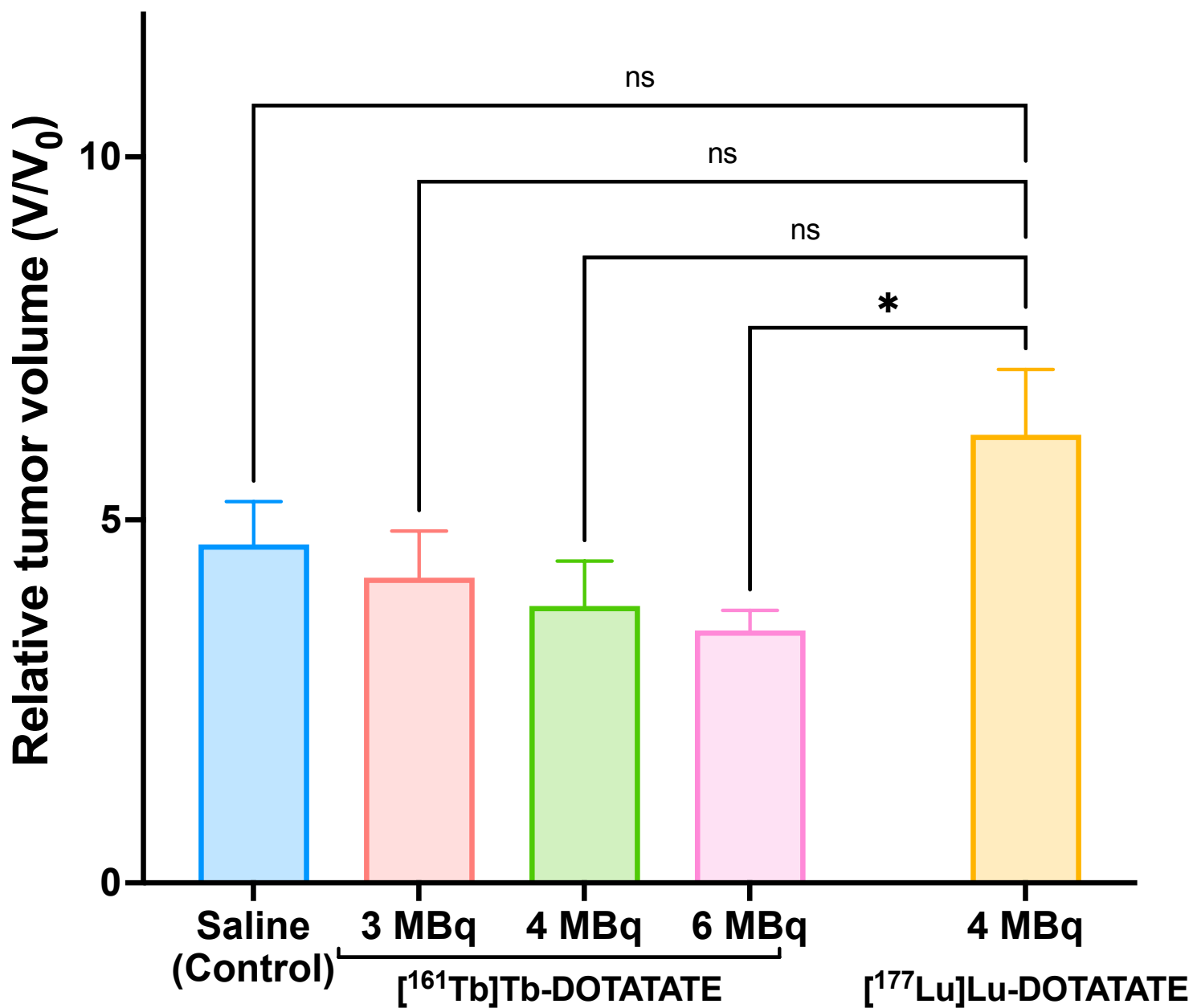

Supplement: Supplementary file 1 — Supplementary Material 1 Bar graph showing baseline-corrected relative tumour volumes on day 16 (n ≥ 7; error bars = SEM). [161Tb]Tb-DOTATATE induced stronger early tumour growth inhibition than [177Lu]Lu-DOTATATE, most pronounced at day 9 (**, p < 0.01). By day 16, tumour growth had however progressed in all groups, and only the 6 MBq [161Tb]Tb-DOTATATE group remained significantly different from the 4 MBq [177Lu]Lu-DOTATATE (*p < 0.05) [file 13550_2026_1487_MOESM1_ESM.pdf]
